# Efficacy and Safety of Roxadustat in Patients with Chronic Kidney Disease: An Updated Meta-Analysis of Randomized Controlled Trials including 6,518 Patients

**DOI:** 10.1155/2022/2413176

**Published:** 2022-11-14

**Authors:** Juanjuan Lei, Han Li, Shixiang Wang

**Affiliations:** Department of Nephrology, Beijing Chao-Yang Hospital, Capital Medical University, Beijing 100020, China

## Abstract

**Background:**

Roxadustat is a newly listed oral hypoxia-inducible factor-proline enhancing enzyme inhibitor (HIF-PHI) in recent years. There have been some studies that have proved the efficacy of roxadustat on the treatment of renal anemia in patients with chronic kidney disease (CKD), but there are still different conclusions on its safety.

**Methods:**

PubMed, Embase, Cochrane, and ClinicalTrials were searched for randomized controlled trials (RCTs) that assess efficacy and safety of roxadustat treatment for anemia in CKD patients. The Cochrane Literature Quality Evaluation Scale was used to evaluate the quality of included literature. We choose fixed-effects model or random effects model for data processing based on heterogeneity. It was considered statistically significant when *p* value <0.05.

**Results:**

A total of 842 articles were retrieved, and 16 trials in the 15 articles were finally included. Roxadustat treatment significantly increased Hb levels. Iron (SMD 1.43, 95% CI 0.31 to 2.55), total iron-binding capacity (SMD 2.06, 95% CI 0.91 to 3.22), ferritin (WMD 21.33, 95% CI 3.04 to 39.62), transferrin saturation (SMD 4.17, 95% CI 3.90 to 4.45), and LDL-cholesterol (SMD -0.64, 95% CI -0.73 to -0.55) showed statistical significance in dialysis-dependent (DD) study. And hepcidin (SMD -1.56, 95% CI -2.63 to -0.50), transferrin (SMD 1.80, 95% CI 1.53 to 2.06), total iron-binding capacity (SMD 1.62, 95% CI 1.39 to 1.86), total cholesterol (SMD -0.88, 95% CI -1.68 to -0.09), ferritin (WMD -52.68, 95% CI -62.68 to -42.67), transferrin saturation (SMD -5.57, 95% CI -7.47 to -3.68), and LDL-cholesterol (SMD -0.85, 95% CI -1.37 to -0.34) showed statistical significance in not dialysis-dependent (NDD) study. In terms of safety, roxadustat treatment did not increase risk of total adverse events either in dialysis-dependent or not dialysis-dependent patients.

**Conclusion:**

Roxadustat can effectively improve anemia in patients with chronic kidney disease. There was no significant difference in total adverse events compared with the control group.

## 1. Introduction

Renal anemia is one of the common complications of patients with chronic kidney disease (CKD). Nearly 90% of long-term dialysis patients have anemia. The lack of erythropoietin and the imbalance of iron metabolism are currently recognized causes of anemia in patients with chronic kidney disease. The use of erythropoiesis stimulators is one of the common methods of clinical treatment of renal anemia. However, under the influence of factors such as iron utilization disorder, chronic inflammation, malnutrition, and low patient compliance, fewer patients use exogenous EPO to treat renal anemia to achieve the goal of hemoglobin [1]. About 10-20% of patients respond poorly to EPO [1].

Roxadustat has been approved for the treatment of renal anemia in some countries as an oral hypoxia-inducible factor-proline hydroxylase inhibitor (HIF-PHI) newly marketed in recent years [2]. Research indicates that the kidneys of CKD patients still retain the ability to produce erythropoietin [3]. Hypoxia-inducible factor (HIF) is a cytokine that contains HIF-*α* (HIF-1*α*, HIF-2*α*, and HIF-3*α*) and HIF-*β* subunits. Under hypoxia, the proline hydroxylase is inactivated, and the concentration of HIF-*α* increases, which promotes the production of EPO by the interstitial cells around the renal tubules [4]. Roxadustat, as a proline hydroxylase inhibitor, can mimic the hypoxic environment, reduce the degradation of HIF, and increase the production of endogenous EPO [5]. A number of clinical studies have confirmed the effectiveness of roxadustat in correcting anemia. Many scholars believe that roxadustat has more adverse reactions in the treatment of renal anemia, mainly involving respiratory tract infections, hypertension, myocardial infarction, hyperkalemia, and gastrointestinal reactions [6]. In the study of Zheng et al., a higher incidence of adverse events (AEs) in the roxadustat group was significantly higher than that in the epoetin alfa group [7]. In recent years, a global phase 3 clinical trial showed that CKD 3-5 patients have a good tolerance to roxadustat [8]. A number of clinical trials have been carried out globally and have obtained new results this year. The results of meta-analyses on the safety of roxadustat are also inconsistent. Related meta-analysis was detailed in the analysis of the efficacy of roxadustat, and the occurrence of specific adverse events was rarely analyzed. We conducted a meta-analysis again including the latest high-quality RCTs to explore the efficacy and safety of roxadustat in the treatment of renal anemia, including the adverse events involved in each study.

## 2. Materials and Methods

### 2.1. Search Strategy

This meta-analysis was conducted to explore the efficacy and safety of roxadustat treatment for anemia in patients with chronic kidney disease (CKD). Our meta-analysis followed the Cochrane Handbook for Systematic Reviews of Interventions and Preferred Reporting for Systematic Review and Meta-Analysis (PRISMA). PubMed, Embase, Cochrane, and ClinicalTrials were searched for studies published through November 2021. We used (((renal dialysis) OR (chronic kidney disease) OR (end-stage kidney disease) OR (ESKD)) AND (roxadustat) AND (anemia)) as the search terms. Moreover, the cited references of included articles and systemic reviews were searched manually.

### 2.2. Selection Criteria

The literature screening was done independently by two authors, and the disagreements between the two authors were determined independently by the third reviewer. Studies that meet the following criteria were included: (1) studies as randomized controlled trials, (2) studies including dialysis patients or patients with stage 3-5 CKD who were not dependent on dialysis, (3) studies evaluating the efficacy and safety of roxadustat in treating anemia, (4) studies reporting the mean change from baseline in efficacy endpoints, and (5) studies reporting adverse events. Experiments where data were not available, nonhuman studies, case reports, systematic reviews, and meta-analysis were excluded. There are no restrictions on gender, race, or region.

### 2.3. Data Extraction

We extracted baseline characteristics such as first author, publication time, country, blinding method, patient and comparator, sample size, and duration from the included studies. Patient inclusion criteria, baseline data, baseline hemoglobin levels, and treatment options were also extracted. The main efficacy outcomes were mean changes from baseline in hemoglobin. Other outcomes included hepcidin, iron, transferrin, soluble transferrin receptor, total iron-binding capacity, total cholesterol, ferritin, transferrin saturation, low-density lipoprotein-cholesterol, triglycerides, and inflammatory markers. We intended to conduct a stratified analysis for hemoglobin levels in different C-reaction protein levels. Adverse events were extracted from the articles to assess the safety. When several articles were published on the same experiment, we selected the latest data.

### 2.4. Statistical Analysis

Statistical analysis was performed in Review Manager 5.3 software. The Cochrane Literature Quality Evaluation Scale was used to evaluate the quality of included literature. We examined heterogeneity by using the *I*^2^ statistics. If the *I*^2^ was >50%, the random effects model was adopted; otherwise, the fixed effects model was adopted. A sensitivity analysis was performed by removing each individual study when showing obvious heterogeneity. It was considered statistically significant when *P* value <0.05. Continuous variables were analyzed by the inverse variance method, and discontinuous variables were analyzed by the Mantel-Haenszel method. The publication bias was evaluated by funnel chart and Egger's test.

Data extraction and statistical analysis were completed by two authors independently, and differences were resolved by a third person.

## 3. Results

### 3.1. Literature Search

**Figure 1:**
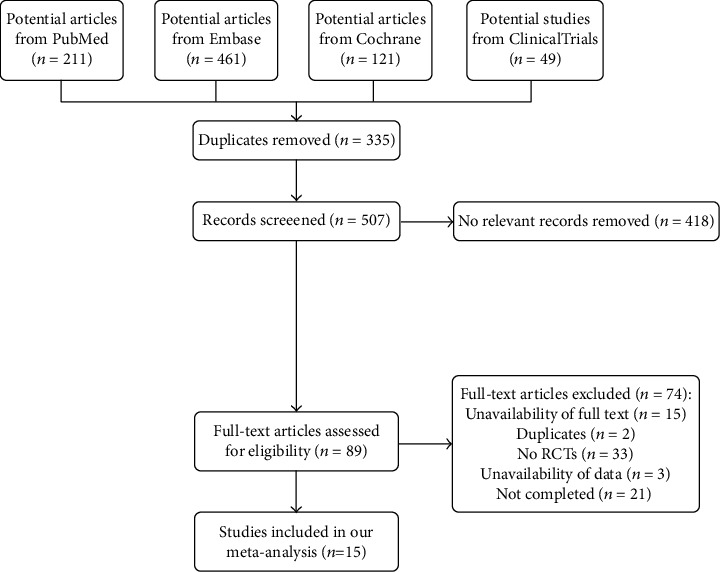
Flow diagram of the literature search and trial selection process.

211 articles were retrieved from PubMed, 461 articles from Embase, 121 articles from the Cochrane, and 49 studies from ClinicalTrials. 335 duplicate documents were excluded. 570 irrelevant articles were excluded after screening titles and abstracts. A total of 89 articles were evaluated for the full text, and 15 articles were included in our meta-analysis finally.  Figure 1 shows the flow chart of the included literature.

### 3.2. Study Characteristics

**Figure 2:**
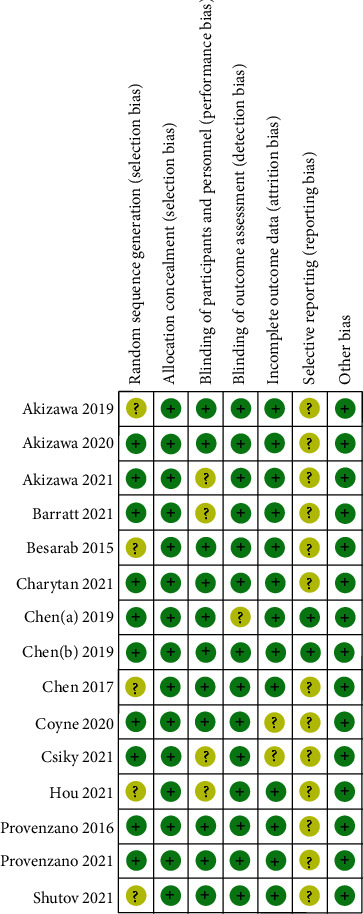
Risk of bias summary for each included study.

The basic characteristics of 16 trials in the 15 articles were shown in Table 1. A total of 8 trials included patients with CKD stage 5 who were dialysis-dependent (DD) and 8 trials included patients who were not dialysis-dependent (NDD) with CKD stage 3–5 patients. The follow-up duration for patients was 6-104 weeks. Each study included 87-1043 patients. The patient baseline variables were similar in the roxadustat group and the control group. Patients had a baseline Hb < 12.0 g/dL. The results of literature quality evaluation were shown in Figure 2.

### 3.3. Efficacy Outcomes of Roxadustat in DD and NDD Study

**Figure 3:**
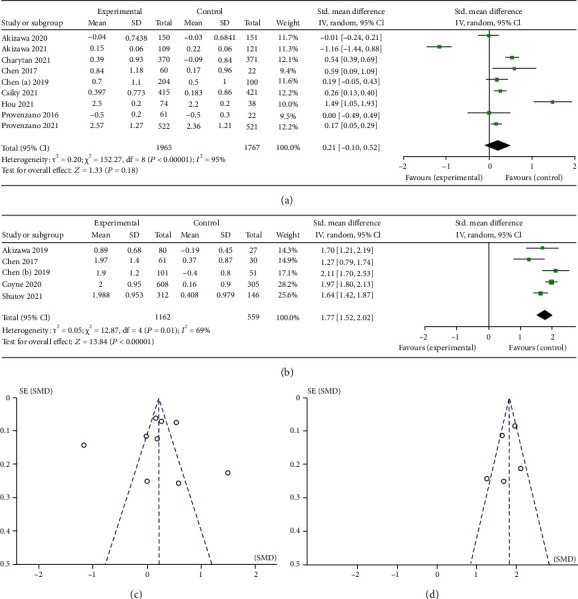
(a) Forest plot of mean change from baseline in Hb level in DD study. (b) Forest plot of mean change from baseline in Hb level in NDD study. (c) Funnel chart of mean change from baseline in Hb level in DD study. (d) Funnel chart of mean change from baseline in Hb level in NDD study.

**Figure 4:**
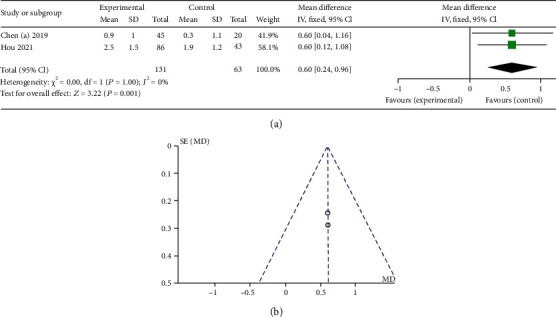
(a) Forest plot of mean change from baseline in Hb level in DD study in patient with a C-reactive protein level above the upper limit of the normal range. (b) Funnel chart of mean change from baseline in Hb level in DD study in patient with a C-reactive protein level above the upper limit of the normal range.

**Figure 5:**
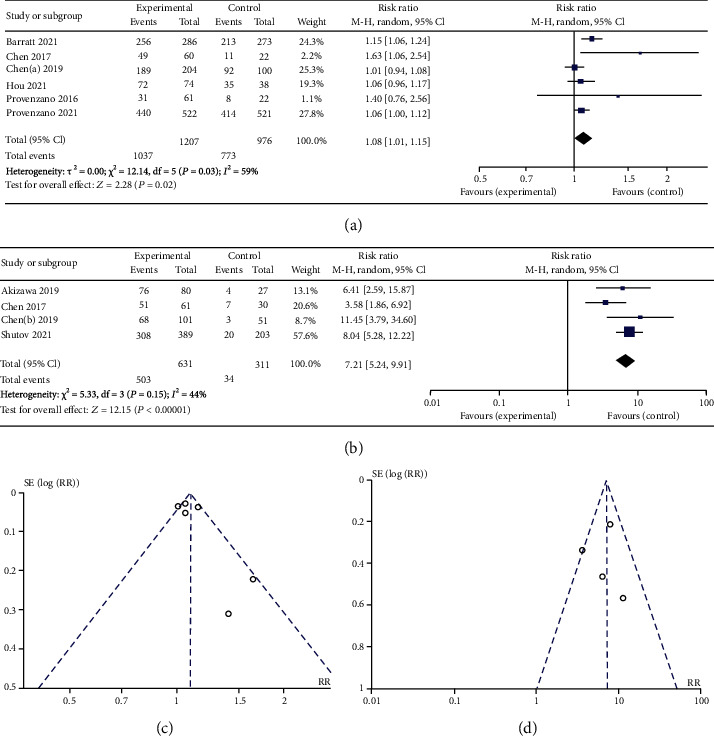
(a) Forest plot of Hb response in DD study. (b) Forest plot of Hb response in NDD study. (c) Funnel plot to assess publication bias in Hb response in DD study. (d) Funnel plot to assess publication bias in Hb response in NDD study.

As shown in Figures 3 and 4, roxadustat treatment significantly increased Hb levels in NDD study (SMD 1.77, 95% CI 1.52 to 2.02, *p* < 0.00001) compared with placebo, and there was no statistical significance in DD study (SMD 0.21, 95% CI -0.10 to 0.52, *p* = 0.18) compared with ESAs. We conducted sensitivity analysis due to obvious heterogeneity. The results showed that there was statistical significance increased Hb levels in DD study after sensitivity analysis (SMD 0.36, 95% CI 0.15 to 0.58, *p* = 0.008). In the analysis that patients with a C-reactive protein (CRP) level above the upper limit of the normal range, roxadustat treatment had a greater Hb levels increased in DD study (WMD 0.60, 95% CI 0.24 to 0.96, *p* = 0.0001). Hb response was defined as an Hb rise not less than 1.0 g/dL from baseline. The forest plots of Hb response in DD study (RR 1.08, 95% CI 1.01 to 1.15, *p* = 0.02) and NDD study (RR 7.21, 95% CI 5.24 to 9.91, *p* < 0.00001) were shown in Figure 5.

As shown in Tables 2 and 3, iron (SMD 1.43, 95% CI 0.31 to 2.55, *p* = 0.01), total iron-binding capacity (SMD 2.06, 95% CI 0.91 to 3.22, *p* = 0.0005), ferritin (WMD 21.33, 95% CI 3.04 to 39.62, *p* = 0.02), transferrin saturation (SMD 4.17, 95% CI 3.90 to 4.45, *p* < 0.00001), and LDL-cholesterol (SMD -0.64, 95% CI -0.73 to -0.55, *p* < 0.00001) showed statistical significance in DD study. And hepcidin (SMD -1.56, 95% CI -2.63 to -0.50, *p* = 0.004), transferrin (SMD 1.80, 95% CI 1.53 to 2.06, *p* < 0.00001), total iron-binding capacity (SMD 1.62, 95% CI 1.39 to 1.86, *p* < 0.00001), total cholesterol (SMD -0.88, 95% CI -1.68 to -0.09, *p* = 0.03), ferritin (WMD -52.68, 95% CI -62.68 to -42.67, *p* < 0.00001), transferrin saturation (SMD -5.57, 95% CI -7.47 to -3.68, *p* < 0.00001), and LDL-cholesterol (SMD -0.85, 95% CI -1.37 to -0.34, *p* = 0.01) showed statistical significance in NDD study. The results showed that there was no change in the conclusion after sensitivity analysis.

**Figure 6:**
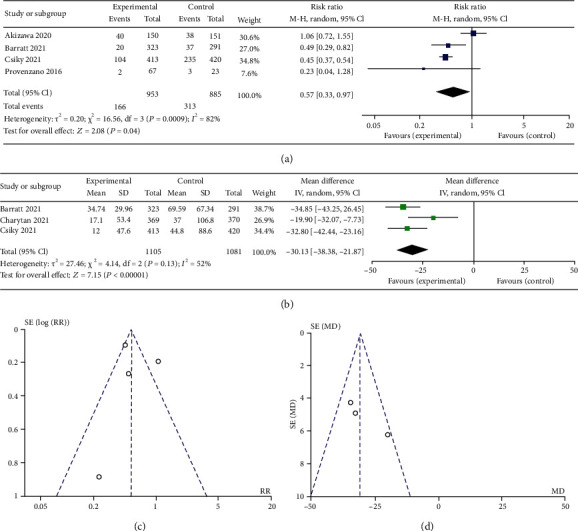
(a) Forest plot of no. of patients using IV iron in DD study. (b) Forest plot of the mean monthly dose IV iron in DD study. (c) Funnel plot of no. of patients using IV iron in DD study. (d) Funnel plot of the mean monthly dose IV iron in DD study.

The analysis of IV iron use in DD study is shown in Figure 6. Compared with ESAs, roxadustat treatment reduced the use (RR 0.52, 95% CI 0.45 to 0.61, *p* = 0.04) and dose (SMD -30.97, 95% CI -36.59 to -25.35, *p* < 0.00001) of IV iron in patients.

### 3.4. Safety Outcomes of Roxadustat in DD and NDD Study

**Figure 7:**
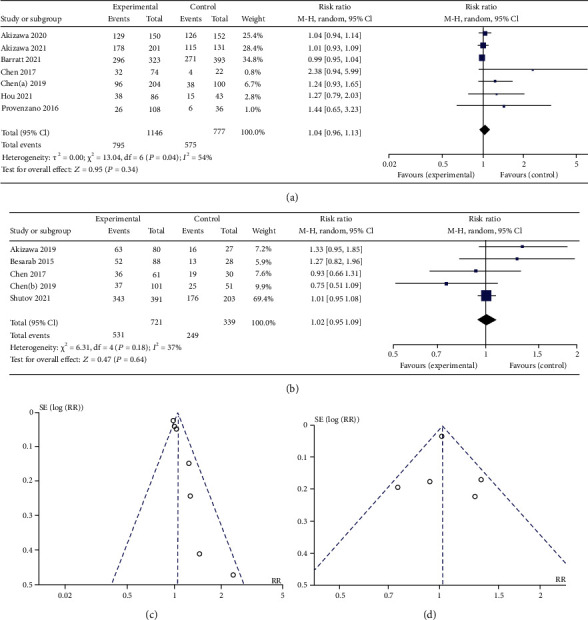
(a) Forest plot of the risk of the main adverse events in DD study. (b) Forest plot of the risk of the main adverse events in NDD study. (c) Funnel plot of the risk of the main adverse events in DD study. (d) Funnel plot of the risk of the main adverse events in NDD study.

As shown in Figure 7 and Tables 4 and 5, there was no significant difference in total adverse events compared with the control group. Compared with ESAs, roxadustat treatment increased the risk of vomiting (RR 1.77, 95% CI 1.08 to 2.90, *p* = 0.02), hypotension (RR 1.45, 95% CI 1.08 to 1.96, *p* = 0.01), diarrhea (RR1.40, 95% CI 1.07 to 1.82, *p* = 0.01), and arteriovenous fistula thrombosis (RR 1.43, 95% CI 1.09 to 1.87, *p* = 0.009) in dialysis patients and reduced the risk of cardiac failure (RR 0.39, 95% CI 0.17 to 0.89, *p* = 0.03). Compared with placebo, roxadustat treatment increased the risk of hypertension (RR 1.45, 95% CI 1.12 to 1.87, *p* = 0.005), hyperkalemia (RR 1.41, 95% CI 1.08 to 1.85, *p* = 0.01), insomnia (RR 3.17, 95% CI 1.66 to 6.07, *p* = 0.0005), nausea (RR 1.79, 95% CI 1.26 to 2.55, *p* = 0.001), and peripheral edema (RR 1.38, 95% CI 1.02 to 1.87, *p* = 0.03) in NDD patients and reduced the risk of anemia (RR 0.18, 95% CI 0.11 to 0.31, *p* < 0.00001).

## 4. Discussion

This meta-analysis included 6,518 patients from 16 trials. We evaluated the efficacy and safety of roxadustat in the treatment of renal anemia. The trials we included are all randomized controlled trials. In our results, roxadustat can effectively improve hemoglobin levels for both dialysis-dependent (DD) and not dialysis-dependent (NDD) patients. Compared with ESAs, roxadustat treatment increased the serum iron level, total iron-binding capacity, and ferritin and reduced the transferrin saturation in patients undergoing dialysis. Compared with placebo, roxadustat treatment reduced the hepcidin, ferritin, and transferrin saturation in NDD patients and increased the transferrin and total iron-binding capacity. In addition, roxadustat can reduce the use of intravenous iron. In the 12 included studies, intravenous iron supplementation was forbidden except for rescue treatment.

We have reached some different conclusions in our research. Compared with previous meta-analyses, we analyzed more adverse events in detail and obtained some new conclusions. Patient in the roxadustat group have an increased risk of vomiting, hypotension, diarrhea, and arteriovenous fistula thrombosis and have a lower risk of cardiac failure and reduced the use of IV iron compared with the ESA group. In NDD study, the risk of hypertension, hyperkalemia, insomnia, nausea, and peripheral edema may increase in the roxadustat group compared with placebo. In our meta-analysis, total adverse events were not statistically significant either in DD or NDD patients. This conclusion is different from the previous meta-analysis results. In the study of Zheng et al., a higher incidence of adverse events (AEs) in the roxadustat group was significantly higher than that in the epoetin alfa group [7].

In our meta-analysis, roxadustat can reduce the level of hepcidin in NDD patients, and it was not statistically significant in DD patients. This is a different result from the previous paper [7, 21]. The inconsistent conclusions may be attributable to short duration and low dose of roxadustat and the dialysis per se [12, 22]. According to Provenzano et al.'s study, significant changes of hepcidin and ferritin were noted in dialysis patients after 19-week treatment of roxadustat [22]. In addition to promoting the production of endogenous EPO, HIF-*α* can also promote the absorption of iron in the intestines and promote the transport of iron in the blood to the tissues [23]. The level of hepcidin is elevated in an inflammatory state, which is currently considered to be one of the reasons for the deficiency of iron utilization [23]. HIF-mediated hypoxia may inhibit the expression of hepatic hepcidin and increase iron utilization [23]. hs-CRP concentration is used as a marker of inflammation. In our results, patients with a C-reactive protein (CRP) level above the upper limit of the normal range, roxadustat treatment had a greater Hb levels increased in DD study. In animal model experiments, the inflammatory response of mice treated with roxadustat was also significantly weakened, which can be demonstrated by the decrease in the infiltration of macrophages and neutrophils and the downregulated expression of inflammatory cytokines [24]. Roxadustat has a positive effect in the treatment of chronic inflammation, and its mechanism is related to the redistribution of oxygen in the cell microenvironment [25]. In the study of Yin et al., the frequency of administration has different effects on inflammation [26]. Due to limited data, we are unable to perform more analysis on inflammation markers in our study. In our meta-analysis, roxadustat can reduce the level of LDL in patients. This may have a protective effect on atherosclerosis [27].

It is generally believed that ESAs will promote platelet function and production results [28]. In 3 studies with a total of 2489 patients, we got the result that roxadustat treatment has an increased risk of arteriovenous fistula thrombosis than ESA treatment. Hypoxia increases the concentration of HIF-1, thereby forming thrombi on atherosclerotic plaques through upregulation of prothrombotic factors [29]. This may be the mechanism that roxadustat treatment increases the risk of arteriovenous fistula thrombosis. A recent animal experiment proved that roxadustat has no effect on platelet production and function in healthy and 5/6 nephrectomized mice [30]. The mechanism is worthy of further exploration.

In our study, roxadustat increased the incidence of hypotension in DD patients and the incidence of hypertension in NDD patients. This is different from the conclusion of Wang et al. [21]. In the study of Yu et al., roxadustat can be used to treat hypertension associated with high renin-angiotensin system (RAS) activity [31]. The mechanism may be through stabilizing HIF-1*α* and then targeting eNOS, AGTR1, AGTR2, and oxidative stress [31]. This may increase the incidence of hypotension. HIF-1*α* and HIF-2*α* play an antagonistic effect in the long-term activation process, which may contribute to the progression of chronic heart failure, atherosclerosis, hypertension, vascular disease, and chronic kidney disease cardiac failure [32]. HIF-2*α* is the main stimulator of erythropoietin synthesis [33]. Roxadustat as an inhibition of isoform-selective prolyl hydroxylases can be achieved in selective activation of HIF-2*α* to ameliorate the development of cardiac failure [32]. Roxadustat increases the levels of HIF-1*α* and HIF-2*α* in CD4+ T cells, reduces their proliferation, and induces apoptosis [34]. Experimental data supports that roxadustat may increase infection by upregulating HIF-1*α* and affecting adaptive immune responses [34], but we did not get the result that roxadustat will increase the infection, either upper respiratory or urinary tract infection. This is the same conclusion as previous studies [21].

There are several limitations in our meta-analysis. (1) The random effects model is used when the heterogeneity is obvious. (2) We have not conducted a subgroup analysis of race, and we cannot rule out its influence. (3) Each study indicated a different initial dose, and we did not control the dose of roxadustat. The observation period of each study was different, which may have some influence on the efficacy and the occurrence of adverse events. The relevant information of inflammation markers is insufficient, and more attention should be paid in future work.

## Figures and Tables

**Table 1:** Baseline characteristics of studies included in the meta-analysis.

Study	Country	Blinded	Patients	Comparator	Sample size	No. of experimental	No. of comparator	Duration (weeks)
Chen et al. [3]	China	Open-label	DD	Epoetin alfa	304	204	100	27
Hou et al. [9]	China	Open-label	PD	ESAs	129	86	43	24
Akizawa et al. [10]	Japan	Double-blind	HD	Darbepoetin	303	151	152	24
Csiky et al. [6]	Europe	Open-label	DD	ESAs	836	415	421	104
Chen et al. [11]	China	Double-blind/open-label	NDD/DD	Placebo/epoetin alfa	91/96	61/74	30/22	8/6
Provenzano et al. [12]	USA	Open-label	HD	Epoetin alfa	144	108	36	19
Provenzano et al. [13]	USA	Open-label	DD	Epoetin alfa	1043	522	521	52
Barratt et al. [14]	UK	Open-label	NDD	Darbepoetin alfa	616	323	293	104
Charytan et al. [15]	USA	Open-label	DD	Epoetin alfa	741	370	371	52
Akizawa et al. [16]	Japan	Open-label	NDD	Darbepoetin alfa	332	201	131	52
Chen et al. [17]	China	Double-blind	NDD	Placebo	152	101	51	8
Shutov et al. [18]	Russia	Double-blind	NDD	Placebo	594	391	203	104
Akizawa et al. [19]	Japan	Double-blind	NDD	Placebo	107	80	27	6
Besarab et al. [20]	USA	Single-blind	NDD	Placebo	116	88	28	12
Coyne et al. [8]	USA	Double-blind	NDD	Placebo	922	616	306	52

DD: dialysis-dependent; NDD: not dialysis-dependent; PD: peritoneal dialysis; HD: hemodialysis.

**Table 2:** Mean change from baseline in iron parameter and lipid levels in DD study.

Parameter	*p* value for heterogeneity (*p*, *I*^2^)	SMD/WMD (95% CI)	*p* value
Hepcidin	*p* = 0.05 *I*^2^ = 63%	-0.12 (-0.39, 0.15)	*p* = 0.38
Iron	*p* < 0.00001 *I*^2^ = 99%	1.43 (0.31, 2.55)	*p* = 0.01
Transferrin	*p* < 0.00001 *I*^2^ = 99%	3.77 (0.04, 7.50)	*p* = 0.05
Soluble transferrin receptor	*p* = 0.009 *I*^2^ = 85%	0.09 (-0.61, 0.79)	*p* = 0.80
Total iron-binding capacity	*p* < 0.00001 *I*^2^ = 99%	2.06 (0.91, 3.22)	*p* = 0.0005
Total cholesterol	*p* < 0.00001 *I*^2^ = 99%	-0.36 (-1.35, 0.62)	*p* = 0.47
Ferritin	*p* = 0.07 *I*^2^ = 53%	21.33 (3.04, 39.62)	*p* = 0.02
Transferrin saturation	*p* = 0.46 *I*^2^ = 0%	4.17 (3.90, 4.45)	*p* < 0.00001
LDL-cholesterol	*p* = 0.20 *I*^2^ = 35%	-0.64 (-0.73, -0.55)	*p* < 0.00001
Triglycerides	*p* = 0.62 *I*^2^ = 0%	-0.09 (-0.39, 0.21)	*p* = 0.56

LDL-cholesterol: low-density lipoprotein cholesterol.

**Table 3:** Mean change from baseline in iron parameter and lipid levels in NDD study.

Parameter	*p* value for heterogeneity (*p*, *I*^2^)	SMD/WMD (95% CI)	*p* value
Hepcidin	*p* < 0.00001 *I*^2^ = 97%	-1.56 (-2.63, -0.50)	*p* = 0.004
Iron	*p* = 0.50 *I*^2^ = 0%	-0.06 (-0.29, 0.18)	*p* = 0.64
Transferrin	*p* = 0.25 *I*^2^ = 27%	1.80 (1.53, 2.06)	*p* < 0.00001
Total iron-binding capacity	*p* = 0.28 *I*^2^ = 21%	1.62 (1.39, 1.86)	*p* < 0.00001
Total cholesterol	*p* = 0.001 *I*^2^ = 90%	-0.88 (-1.68, -0.09)	*p* = 0.03
Ferritin	*p* = 0.13 *I*^2^ = 47%	-52.68 (-62.68, -42.67)	*p* < 0.00001
Transferrin saturation	*p* = 0.30 *I*^2^ = 18%	-5.57 (-7.47, -3.68)	*p* < 0.00001
LDL-cholesterol	*p* = 0.03 *I*^2^ = 78%	-0.85 (-1.37, -0.34)	*p* = 0.01

LDL-cholesterol: low-density lipoprotein cholesterol.

**Table 4:** TEAEs occurring in the treatment groups in DD study.

Parameter	*p* value for heterogeneity (*p*, *I*^2^)	RR (95% CI)	*p* value
TEAEs	*p* = 0.04, *I*^2^ = 54%	1.04 (0.96, 1.13)	*p* = 0.34
Serious TEAEs	*p* = 0.68, *I*^2^ = 0%	1.09 (0.97, 1.23)	*p* = 0.14
Drug-related serious TEAEs	*p* = 0.63, *I*^2^ = 0%	1.10 (0.96, 1.26)	*p* = 0.18
Upper respiratory tract infection	*p* = 0.37, *I*^2^ = 7%	1.07 (0.70, 1.64)	*p* = 0.76
Urinary tract infection	*p* = 0.71, *I*^2^ = 0%	1.76 (0.71, 4.40)	*p* = 0.22
Pneumonia	*p* = 0.63, *I*^2^ = 0%	1.03 (0.77, 1.37)	*p* = 0.86
Hypertension	*p* = 0.71, *I*^2^ = 0%	1.13 (0.93, 1.37)	*p* = 0.21
Hypertensive crisis	*p* = 0.79, *I*^2^ = 0%	0.79 (0.39, 1.60)	*p* = 0.52
Hyperkalemia	*p* = 0.11, *I*^2^ = 44%	1.03 (0.80, 1.33)	*p* = 0.83
Headache	*p* = 0.43, *I*^2^ = 0%	1.22 (0.92, 1.62)	*p* = 0.17
Chest discomfort	*p* = 0.13, *I*^2^ = 56%	3.31 (0.21, 52.28)	*p* = 0.39
Vomiting	*p* = 0.44, *I*^2^ = 0%	1.77 (1.08, 2.90)	*p* = 0.02
Nausea	*p* = 0.02, *I*^2^ = 56%	1.48 (0.83, 2.65)	*p* = 0.19
Asthenia	*p* = 0.45, *I*^2^ = 0%	2.30 (0.67, 7.87)	*p* = 0.19
Alanine aminotransferase increase	*p* = 0.93, *I*^2^ = 0%	1.48 (0.55, 3.98)	*p* = 0.43
Dizziness	*p* = 0.65, *I*^2^ = 0%	1.02 (0.65, 1.60)	*p* = 0.95
Hypotension	*p* = 0.71, *I*^2^ = 0%	1.45 (1.08, 1.96)	*p* = 0.01
Muscle spasms	*p* = 0.004, *I*^2^ = 74%	0.63 (0.28, 1.39)	*p* = 0.25
Anemia	*p* = 0.26, *I*^2^ = 26%	1.07 (0.63, 1.83)	*p* = 0.80
Atrial fibrillation	*p* = 0.97, *I*^2^ = 0%	0.81 (0.48, 1.37)	*p* = 0.43
Diarrhea	*p* = 0.13, *I*^2^ = 47%	1.40 (1.07, 1.82)	*p* = 0.01
Constipation	*p* = 1.00, *I*^2^ = 0%	1.51 (0.91, 2.50)	*p* = 0.11
Pruritus	*p* = 0.94, *I*^2^ = 26%	1.36 (0.80, 2.30)	*p* = 0.26
Peritonitis	*p* = 0.58, *I*^2^ = 0%	0.82 (0.40, 1.66)	*p* = 0.58
Hyperparathyroidism secondary	*p* = 0.32, *I*^2^ = 0%	1.09 (0.73, 1.64)	*p* = 0.66
Injury, poisoning, and procedural complications	*p* = 0.75, *I*^2^ = 0%	0.87 (0.68, 1.11)	*p* = 0.27
Arteriovenous fistula thrombosis	*p* = 0.66, *I*^2^ = 0%	1.43 (1.09, 1.87)	*p* = 0.009
Coronary artery disease	*p* = 0.62, *I*^2^ = 0%	0.22 (0.04, 1.35)	*p* = 0.10
Acute myocardial infarction	*p* = 0.78, *I*^2^ = 0%	0.59 (0.29, 1.21)	*p* = 0.15
Cardiac failure	*p* = 0.32, *I*^2^ = 2%	0.39 (0.17, 0.89)	*p* = 0.03
Gastroenteritis	*p* = 0.72, *I*^2^ = 0%	1.06 (0.26, 4.29)	*p* = 0.94
Pancreatitis	*p* = 0.27, *I*^2^ = 19%	4.99 (0.73, 34.09)	*p* = 0.10
Cellulitis	*p* = 0.89, *I*^2^ = 0%	0.82 (0.25, 2.75)	*p* = 0.75
Sepsis	*p* = 0.47, *I*^2^ = 0%	1.12 (0.60, 2.09)	*p* = 0.73
Gangrene	*p* = 0.26, *I*^2^ = 27%	1.34 (0.54, 3.29)	*p* = 0.53

TEAEs: treatment emerged adverse event.

**Table 5:** TEAEs occurring in the treatment groups in NDD study.

Parameter	*p* value for heterogeneity (*p*, *I*^2^)	RR (95% CI)	*p* value
TEAEs	*p* = 0.18, *I*^2^ = 37%	1.02 (0.95, 1.09)	*p* = 0.64
Serious TEAEs	*p* = 0.56, *I*^2^ = 0%	1.09 (0.94, 1.25)	*p* = 0.26
ESKD	*p* = 0.08, *I*^2^ = %	1.38 (0.85, 2.25)	*p* = 0.19
Upper respiratory tract infection	*p* = 0.76, *I*^2^ = 0%	0.79 (0.58, 1.08)	*p* = 0.14
Urinary tract infection	*p* = 0.44, *I*^2^ = 77%	0.46 (0.05, 4.55)	*p* = 0.50
Cough	*p* = 0.14, *I*^2^ = 54%	0.61 (0.13, 2.98)	*p* = 0.54
Pneumonia	*p* = 0.70, *I*^2^ = 0%	1.14 (0.76, 1.71)	*p* = 0.52
Viral upper respiratory tract infection	*p* = 0.14, *I*^2^ = 53%	1.50 (0.87, 2.61)	*p* = 0.15
Nasopharyngitis	*p* = 0.63, *I*^2^ = 0%	1.27 (0.59, 2.75)	*p* = 0.54
Hypertension	*p* = 0.72, *I*^2^ = 0%	1.45 (1.12, 1.87)	*p* = 0.005
Hyperkalemia	*p* = 0.94, *I*^2^ = 0%	1.41 (1.08, 1.85)	*p* = 0.01
Metabolic acidosis	*p* = 0.05, *I*^2^ = 74%	1.77 (0.24, 13.04)	*p* = 0.58
Insomnia	*p* = 0.17, *I*^2^ = 46%	3.17 (1.66, 6.07)	*p* = 0.0005
Gout	*p* = 0.18, *I*^2^ = 43%	0.72 (0.43, 1.20)	*p* = 0.21
Back pain	*p* = 0.04, *I*^2^ = 75%	0.47 (0.03, 8.90)	*p* = 0.62
Headache	*p* = 0.88, *I*^2^ = 0%	1.16 (0.82, 1.66)	*p* = 0.40
Vomiting	*p* = 0.69, *I*^2^ = 0%	1.38 (0.85, 2.24)	*p* = 0.20
Nausea	*p* = 0.38, *I*^2^ = 3%	1.79 (1.26, 2.55)	*p* = 0.001
Dizziness	*p* = 0.22, *I*^2^ = 32%	0.77 (0.53, 1.11)	*p* = 0.16
Muscle spasms	*p* = 0.12, *I*^2^ = 53%	1.04 (0.20, 5.44)	*p* = 0.96
Anemia	*p* = 0.52, *I*^2^ = 0%	0.18 (0.11, 0.31)	*p* < 0.00001
Diarrhea	*p* = 0.17, *I*^2^ = 38%	1.38 (1.00, 1.92)	*p* = 0.05
Peripheral edema	*p* = 0.71, *I*^2^ = 0%	1.38 (1.02, 1.87)	*p* = 0.03
Fever	*p* = 0.05, *I*^2^ = 73%	1.02 (0.17, 6.12)	*p* = 0.98
Pruritus	*p* = 0.09, *I*^2^ = 65%	2.28 (0.65, 7.93)	*p* = 0.20
Asthenia	*p* = 0.63, *I*^2^ = 0%	1.48 (0.77, 2.85)	*p* = 0.24
Gastrointestinal hemorrhage	*p* = 0.89, *I*^2^ = 0%	0.82 (0.25, 2.75)	*p* = 0.75

TEAEs: treatment emerged adverse event.

## Data Availability

All data are available from the corresponding author.
